# Influence of salinity on growth, nutrient utilization, and gene expression of giant freshwater prawn (*Macrobrachium rosenbergii*)

**DOI:** 10.1371/journal.pone.0353240

**Published:** 2026-07-23

**Authors:** Farhana Ahamed Shanila, Md. Rajib Sharker, Md. Rafiqul Islam, Niels O. G. Jørgensen, Mohammad Lokman Ali

**Affiliations:** 1 Department of Aquaculture, Faculty of Fisheries, Patuakhali Science and Technology University, Patuakhali, Bangladesh; 2 Department of Fisheries Biology and Genetics, Faculty of Fisheries, Patuakhali Science and Technology University, Patuakhali, Bangladesh; 3 Department of Plant and Environmental Sciences, University of Copenhagen, Frederiksberg, Denmark; Shanghai Ocean University, CHINA

## Abstract

Salinity is one of the most influential eco-hydrological factors that affect the molting, growth, and other physico-biochemical responses of crustaceans. A 56-day in vivo experiment was conducted to assess the effects of variable salinities on growth attributes, feed utilization, and gene expression profile of Macrobrachium rosenbergii. Juveniles of M. rosenbergii that were exposed to salinities of 0, 3, 6, 9, and 12 ppt in triplicate, and designated as control, T1, T2, T3, and T4, respectively. Higher weight gain (WG), %WG, specific growth rate (SGR), feed conversion ratio (FCR), and protein efficiency ratio (PER) were recorded at 3 ppt than at other salinities. A salinity of 3 ppt also showed higher values of condition factor (CF), and survival of *M. rosenbergii*. A polynomial regression analysis indicated that the optimal salinity for WG and SGR of *M. rosenbergii* were 3.12 and 3.80, respectively. A significant decrease in the molt-inhibiting hormone (MIH) and an increase in the chitinase (CHIT) gene expression were documented at 3 ppt compared to control. The mRNA transcripts of stress response genes were significantly downregulated, and immune-related genes were upregulated at 3 ppt salinity, compared to the other salinities. Although the expression of antioxidant-related genes was upregulated when rearing at 3 ppt salinity, no significant differences were found relative to other salinities. Our findings suggest that *M. rosenbergii* reared at 3 ppt salinity show promising improvements in growth attributes by modulating the expression profile of physico-biochemical response genes reared at low temperature.

## 1. Introduction

Salinity is one of the key environmental variables influencing growth, reproduction, survival, and distribution of aquatic invertebrates [1,2]. Although many crustaceans exhibit a wide tolerance to salinity fluctuations, the optimal salinity range for individual species is specific, varying with physiological adaptations and ecological requirements [3–5]. In recent years, climate variability has caused rising sea levels, coastal flooding, and intensified tropical cyclones, leading to salinity-induced water stress in freshwater fisheries in various regions of the world [6]. In freshwater species, salinity fluctuations affect the physiology, reproductive performance, abundance, and distribution [7–9]. Salinity also has a strong influence on embryonic and larval development, growth, and production of aquaculture species [10,11]. In aquaculture, salinization of the water has a major impact on the sustainability of the production by modifying the feed utilization, metabolic activity, immune performance and survival of farmed species [12–14]. Saltwater intrusion during flooding in southern Bangladesh raises salinity levels in shallow prawn farms, causing stress, reduced growth, and high mortality in freshwater prawn *M. rosenbergii* [15].

Among aquatic crustaceans, several species have been documented to rear in inland saline waters across various parts of the globe [16,17]. Salinity intrusion poses a significant challenge to freshwater aquaculture due to severe physiological stress that might eventually lead to their extinction due to inability to adapt such harsh condition. Thus, it is crucial to determine the optimal salinity level for economically significant prawn species in aqua-farming systems, where salinity can be adjusted to suit the species growth, survival, and overall productivity.

Globally, the giant freshwater prawn (*M. rosenbergii*) is an economically significant crustacean species owing to its fast growth and tolerance to various environmental stressors, as well as disease resistance, delicate carcass quality, and high market value compared to other shellfish species [18]. *M. rosenbergii* is rich in proteins, essential amino acids, and polyunsaturated fatty acids, while being low in fat, making this prawn a cherished and nutritious food preference for human consumption [19]. *M. rosenbergii* production is more economically and environmentally sustainable than other intensively reared crustacean species due to its use of low stocking densities [20].

Molecular tools are essential in aquaculture research, complementing traditional methods and enhancing insights into fish and shellfish responses to culture stress and environmental fluctuations [21–23]. Gene expression assays play a central role in understanding the molecular and physiological responses of aquatic organisms to salinity changes [24]. By analyzing the expression of genes related to growth, oxidative stress, and immunity, researchers can decipher how organisms maintain cellular homeostasis under varying salinity conditions [25].

The influence of salinity on the survival and growth has been reported for many economically significant penaeids [26–28]. Previous investigations demonstrated the effects of salinity gradients on physiological, biochemical and genetic markers in the prawn [4, 29, 30]. Osmoregulation, growth and molting cycles of *M. rosenbergii* in the Mekong river delta were found to be influenced by different salinity level [31]. Elevated salinity negatively affected the reproduction and growth of female *M. Rosenbergii* [32], and the highest growth rate was documented at 10 ppt salinity [33].

However, a few studies have investigated the effect of salinity on growth, and survival of *M. rosenbergii*, and changes in gene expression profiles at different salinities have not yet been studied. To address these issues, the present study was carried out to examine growth performance, feed utilization efficacy, survival, and mRNA expression of genes involved in growth, stress, and immune response at different salinities.

## 2. Materials and methods

### 2.1. Collection and acclimatization of experimental species

Juveniles of *M. rosenbergii* were procured from a commercial hatchery (Patuakhali, Bangladesh) and transported into an oxygenated polythene bag. The experiment was conducted at the facilities of the wet laboratory, Department of Aquaculture, Patuakhali Science and Technology University. Before the commencement of the experimental trial, *M. rosenbergii* were acclimatized under controlled laboratory condition for two weeks, and fed a commercial diet and live food up to the ad libitum.

### 2.2. Experimental layout

**Fig 1:**
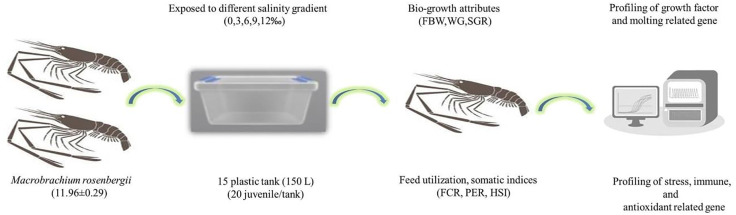
The experimental layout of salinity-induced changes in growth, feed utilization, and gene expression of *M.* rosenbergii.

After the acclimatization period, active and healthy juveniles of *M. rosenbergii* were randomly allocated into five experimental categories. *M. rosenbergii* were equally distributed in 15 plastic tanks, and exposed to different salinity levels (0, 3, 6, 9, and 12 ppt) for 56 days (Fig 1). Three biological replications (20 juveniles/replication) for each experimental treatment were applied to conduct this study, and referred to as control, T1, T2, T3, and T4, respectively. Sufficient aeration was maintained using mechanical aerator and fed live food and commercial pelleted (35% crude protein, moisture 11%, lipid 6%, carbohydrate 22%, crude fiber 5%, ash 10%, calcium 2.5%, phosphorus 1.2%) feed twice a day up to the satiation level. Siphoning was done to remove the leftover food and fecal matters daily.

### 2.3. Growth performance, feed utilization, and survival

Following the experimental trial, *M. rosenbergii* were subjected to a one-day starvation period. Subsequently, the total length and final weight of *M. rosenbergii* were measured by using a slide calipers, and a digital balance, respectively. Growth parameters (weight gain, percentage weight gain, specific growth rate, survival), feed utilization parameters (feed conversion ratio, protein efficiency ratio), biometric indices (hepato-somatic index, meat ratio), and condition factor were calculated by using formula as reported by [34].


$$\begin{array}{c} \text{Weight}\;\text{gain}\;\left(\text{g}\right)=\text{Final}\;\text{weight}\;-\text{initial}\;\text{weight}\; \end{array}$$

$$\begin{array}{c} \text{Weight}\;\text{gain}\;(\%)=\frac{\text{Final}\;\text{weight}-\;\text{initial}\;\text{weight}}{\text{Initial weight}}\times \text{100} \end{array}$$

$$\begin{array}{c} \text{Specific}\;\text{growth}\;\text{rate}\;(\text{SGR},\%/\text{day})=\frac{\text{LN}\;\text{final}\;\text{weight}-\text{Ln}\;\text{initial}\;\text{weight}}{\text{Number}\;\text{of}\;\text{days}}\times \text{100} \end{array}$$

$$\begin{array}{c} \text{Feed}\;\text{conversion}\;\text{ratio}\;(\text{FCR})=\frac{\text{Total}\;\text{feed}\;\text{intake}}{\text{Weight}\;\text{gain}} \end{array}$$

$$\begin{array}{c} \text{Protein}\;\text{efficiency}\;\text{ratio}\;(\text{PER})=\frac{\text{Weight}\;\text{gain}}{\text{Crude}\;\text{protein}\;\text{fed}} \end{array}$$

$$\begin{array}{c} \text{C}\text{ondition}\;\text{factor}\;(\text{C}\text{F})=\text{Weight}/{\text{Length}}^{\text{3}}\times \text{100} \end{array}$$

$$\begin{array}{c} \text{Survival}\;\text{rate}\;(\%)=\frac{\text{Number}\;\text{of}\;\text{fish}\;\text{harvested}}{\text{Number}\;\text{of}\;\text{fish}\;\text{stocked}}\times \text{100} \end{array}$$

$$\begin{array}{c} \text{Hepato}-\text{somatic index }=\frac{\text{Hepatic weight}}{\text{Body weight}}\times \text{100} \end{array}$$

$$\begin{array}{c} \text{Meat ratio }=\frac{\text{Edible flesh weight}}{\text{Body weight}}\times \text{100} \end{array}$$

### 2.4. Profiling of gene expression

Expression of the following genes, assumed important to growth, stress and immune response in *M. rosenbergii,* was studied: CHH (crustacean hyperglycemic hormone), MSTN (myostatin), CHIT3 (Chitinase 3), MIH (molt-inhibiting hormone), HSP 60 (heat shock protein 60), HSP 90 (heat shock protein 90), Cu/Zn SOD (superoxide dismutase), CAT (Catalase); α2-M, (alpha-2-macroglobulin); NF-κB (nuclear factor kappa B) The primers used for gene expression analysis are listed in **Table 1**. Hepatopancreas tissue samples were selected from each replication for the analysis of gene expression due to its role in metabolism, detoxification, and immunity. qRT-PCR assayfor the different genes were done following the method by [35–38]. Briefly, total RNA was extracted using an RNeasy mini kit (Qiagen, Hilden, Germany), after which Invitrogen’s Superscript® III First-Strand synthesis kit (Carlsbad, CA, USA) was employed for cDNA synthesis following the kit´s manual. The β-actin gene was chosen as an internal control, and three biological replicates were used for the qRT-PCR assays. The relative gene expression was quantified using the 2^−ΔΔct^ method based on the cycle threshold [39].

**Table 1:** Forward (F) and reverse (R) primers used for real-time quantitative RT-PCR.

Name	Accession number	Primer sequence: 5′-3′	Amplicon size	Tm (ºC)	Reference
*CHH*	AF219382.1	F: CAGGTTCTTTTTCCCCCTTT	202	56	[74]
		R: ATCAACGCGAAAGCCTCAT		55	
*MSTN*	JQ283113.1	F: ACTGCGCTGTGTTGATTGTAGCTG	189	57	[75]
		R: ACAACAGTACGTGTTCACGGGTCT		57	
*MIH*	AF432346.1	F: GTAAACCAGACAACGCAAGGG	194	56	[76]
		R: TTCATCCGGAAGATATTGGA		54	
*CHIT3*	LT574899	F:GGGCTTGGCTGGTTGTAT	193	56	[77]
		R:GGTGGAGGTGGAGTTGGA		58	
*HSP60*	KC521465	F: CGACGCCAACGGAATCCTAAAT	196	62	[78]
		R: CTTTGCTCAGTCTGCCCTTGT		61	
*HSP90*	GU319963.1	F: GAAGGAAAGGGACAAGGA	238	54	[79]
		R: GGTCCATAAAGGCTTGGT		54	
*Cu/Zn SOD*	KF590042	F: GGAGCTAGATTGGCTTGCTG	182	60	[80]
		R: AGAAAATCACTGCCCCTCCT		58	
*CAT*	HQ668089.1	F: ACTACAACCAGGAAAGTGCTCCCA	205	57	[80]
		R: TGGCGTTCCTCTTCGTTCATGACT		57	
*α2-M*	ABK60046	F:CTCGGCCATCTTATCCGTATG	184	61	[81]
		R:GGGAGCGAAGTTGAGCATGT		60	
*NF-κB*	XM_067095087.1	F:AGATGCCGAGGAGGTATGGA	191	60	[82]
		R:GCGTCGTTGAAATGCGATGT		58	
*β-actin*	GQ131934	F: CTGTTACGGGTGACGGAGAA	150	60	[77]
		R: TCGGAAGAGTCCCGCATT		56	

CHH, Crustacean hyperglycemic hormone; MSTN, Myostatin; CHIT3, Chitinase 3; MIH, Molt-inhibiting hormone HSP 60, Heat shock protein 60; HSP 90, Heat shock protein 90; Cu/Zn SOD, superoxide dismutase; CAT, Catalase; α2-M, Alpha-2-macroglobulin; NF-κB, Nuclear factor kappa B

### 2.5. Statistical analysis

The data were analyzed using one-way ANOVA in GraphPad Prism 9. Duncan's multiple range tests was used to detect significant differences among different treated groups at a 95% confidence level. All values are presented as mean ± standard deviation, with significance set at P ≤ 0.05. Biometric indices, condition factor, and survival were analyzed and visualized using the open-source R environment (version 4.2.1). The optimal salinity level for WG (g) and SGR (%/day) in *M. rosenbergii* was estimated using second-order polynomial regression analysis. The identified break–point indicates the optimum salinity level for maximizing growth. All the data in different treatment groups are presented as mean ± SD.

## 3. Results

### 3.1. Water quality parameters

The physico-chemical attributes of tank water measured during the experimental period were presented in **Table 2**. Throughout the trial period, these parameters did not significantly change or affect the different salinity treatments.

**Table 2:** Analyzed water quality parameters following a 56-days growth trial of *M. rosenbergii* under different salinities. All calculated values are expressed as Mean±SD.

Parameters	Treatment				
	T1	T2	T3	T4	T5
Temperature (℃)	17.7 ± 1.94	17.4 ± 2,48	17.9 ± 1.98	17.5 ± 2.18	17.2 ± 2.83
pH	7.3 ± 1.16	7.25 ± 1.74	7.38 ± 1.08	7.41 ± 0.1.63	7.43 ± 0.58
Dissolved oxygen (ppm)	6.24 ± 0.43	6.87 ± 0.23	6.67 ± 0.23	6.63 ± 0.58	6.34 ± 0.43
Alkalinity(ppm)	117 ± 51.21	120.67 ± 89.43	108.3 ± 87.33	119.3 ± 81.37	115.33 ± 56.44
Ammonia(ppm)	0.15 ± 0.08	0.17 ± 0.05	0.18 ± 0.08	0.15 ± 0.06	0.25 ± 0.08
Nitrite(ppm)	0.06 ± 0.0.01	0.08 ± 0.04	0.07 ± 0.09	0.12 ± 0.34	0.10 ± 0.14
Salinity(ppt)	0 (control)	3	6	9	12

### 3.2. Growth, and feed utilization attributes

**Fig 2:**
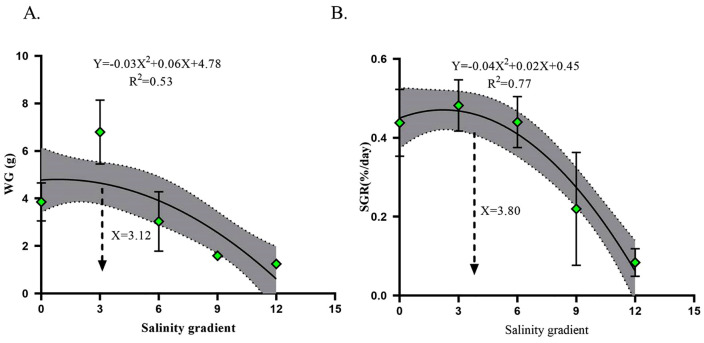
Second-order polynomial regression analysis of A. WG (g), (B) SGR (%/day) in *M*. *rosenbergii* across different salinities over 56-days.

The initial mean body weights of the *M. rosenbergii* were not significantly different (P > 0.05) at the beginning of the experimental trial. At the end of the 56-day experimental duration, the growth exhibited a significant increase in all examined growth metrices at 3 ppt as compared to the control and other treatments. Highest weight gain (WG), % WG, and specific growth rate (SGR) were recorded in prawns cultured in 3 ppt, followed by 0, 6, 9, and 12 ppt (Table 3). The quadratic regression analysis based on the WG (g) and SGR (%/day) demonstrated that the optimum salinity for the highest growth performance of *M. rosenbergii* was 3.12 for WG and 3.80 for SGR (Fig 2). The prawns reared at 3 ppt also demonstrated the best feed conversion ratio (FCR) compared to the other treatments. The lowest value of protein efficiency ratio (PER) was obtained in prawns cultured in 12 ppt (Table 3).

**Table 3:** Growth attributes for *M. rosenbergii* reared in different salinities over a 56-day period. All calculated values are expressed as Mean±SD.

Attributes	Salinity (ppt)				
	0 (control)	3	6	9	12
IBW (g)	11.96 ± 1.12	11.91 ± 0.62	11.85 ± 1.78	11.95 ± 2.12	11.97 ± 0.97
FBW (g)	15.40 ± 0.15^a^	17.68 ± 0.46^b^	15.21 ± 0.43^a^	13.52 ± 1.24^c^	13.20 ± 0.46^c^
WG (g)	3.44 ± 0.15^a^	5.77 ± 0.46^b^	3.36 ± 0.43^a^	1.57 ± 1.24^c^	1.23 ± 0.46^b^
% WG (g)	28.76 ± 1.28^a^	48.45 ± 3.91^b^	28.35 ± 3.56^a^	11.62 ± 2.83^c^	10.31 ± 3.91^d^
SGR (%/day)	0.42 ± o.o8^a^	0.53 ± 0.23^b^	0.41 ± 0.04^a^	0.21 ± 0.08^d^	0.16 ± 0.04^c^
FCR	1.8 ± 0.15^a^	1.05 ± 0.43^b^	1.71 ± 0.54^a^	2.15 ± 0.58^c^	2.18 ± 0.62^c^
PER	2.13 ± 0.46^a^	2.84 ± 0.85^b^	2.10 ± 0.39^a^	1.72 ± 0.58^c^	1.68 ± 0.39^c^

Different superscript letters in each row indicate a significant difference (*P* < 0.05). IBW: Initial body weight; FBW: Final body weight; WG: Weight gain; SGR: Specific growth rate; FCR: Feed conversion ratio.

### 3.3. Biological indices, condition factor, and survival

**Fig 3:**
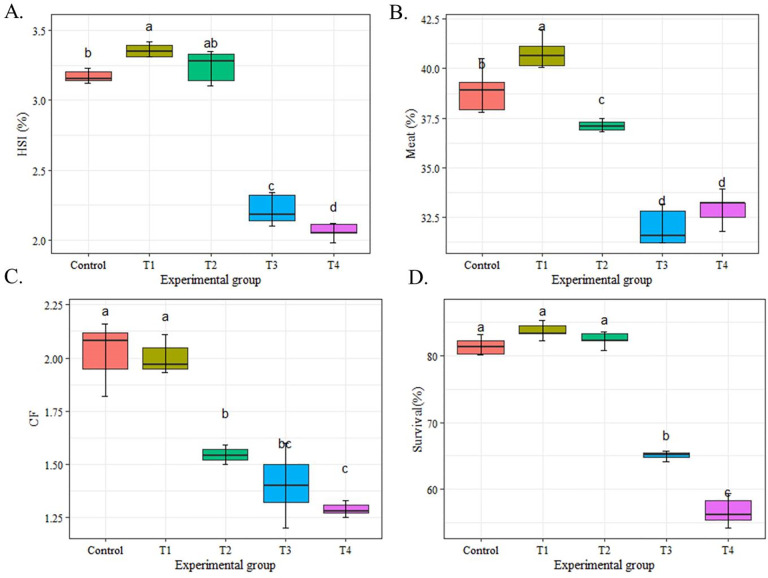
Assessment of biometric indices, condition factor, and survival rate of M. rosenbergii subjected to varying salinity regimes.

Biological indices, condition factor, and survival of *M. rosenbergii* under different salinity treatments are presented in **Fig 3**. The highest values of hepatosomatic index (HSI) and meat ratio were determined in the T1 (3ppt) treatment group. The maximum condition factor (CF) was found in the control group, however not significantly different from the T1 group. The survival was similar between the control, T1, and T2 groups (P > 0.05) but lower for the T3 and T4 groups (P < 0.05)

### 3.4. Profiling of gene expression

The expression of genes involved in growth, stress response, antioxidant enzymes, and immunity were studied under different salinity treatments. The quantity of total RNA isolated from the hepatopancreas of the prawns is presented in **Supplementary Table 1**. In the control treatment, the total RNA level was about 750.2 ± 1.63 ng/µL. The prawns reared in 3ppt salinity exhibited a higher amount of total RNA (910.8 ± 2.13 ng/µL) than did other tested treatments.

### 3.5. Changes in expression of growth factor gene

**Fig 4:**
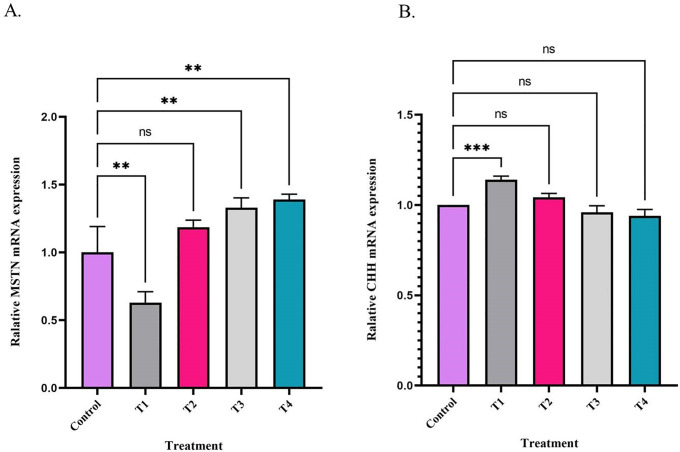
Changes in relative mRNA transcript of growth factor-related genes in hepatopancreas (A), MSTN (B), CHH of *M. rosenbergii* after exposure to different salinities.

The expression levels of MSTN and CHH transcript in *M. rosenbergii* are shown in **Fig 4**. The mRNA expression of these genes was influenced by the different salinity treatments. *M. rosenbergii* exposed to a rearing salinity of 3 ppt exhibited a significantly lower MSTN mRNA transcript level as compared to the control group. However, when the rearing salinity was further increased to 6 ppt, no significant differences in mRNA transcript levels were observed relative to the control. Significantly higher expression was found when rearing at 9 ppt and 12 ppt (**Fig 4A**). The transcript of CHH was significantly upregulated when rearing at 3 ppt. Although CHH mRNA expression showed a moderate increase at 6 ppt, the change was not statistically significant (Fig 4B). No significant difference in mRNA transcript levels was observed among exposures at 0, 9, and 12.

### 3.6. Changes in expression of molting-related gene

**Fig 5:**
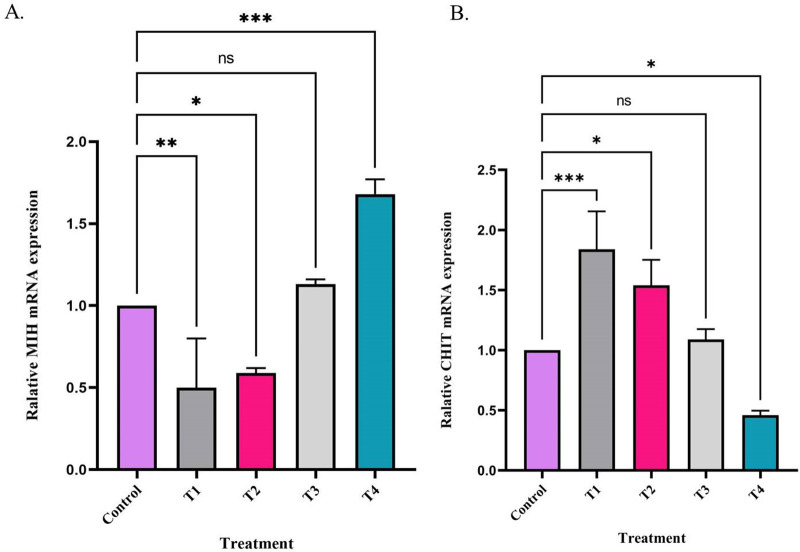
Changes in relative mRNA transcript of molting related gene in hepatopancreas of (A), MIH (B), CHIT of M. rosenbergii after exposure to different salinities.

The expressions of molting-related genes (MIH and CHIT) were modulated under different salinity treatments in *M. rosenbergii* (**Fig 5**). At 3 ppt salinity, the MIH gene expression was down-regulated markedly, whereas the CHIT was up-regulated, as compared to the control (**Fig 5A**, **5B**). The expression levels of the MIH and CHIT genes were elevated at 9 ppt but showed no significant difference compared to the control. Significantly higher expression of MIH and lower expression of CHIT was found in the 12 ppt salinity treatment group.

### 3.7. Changes in expression of stress response gene

**Fig 6:**
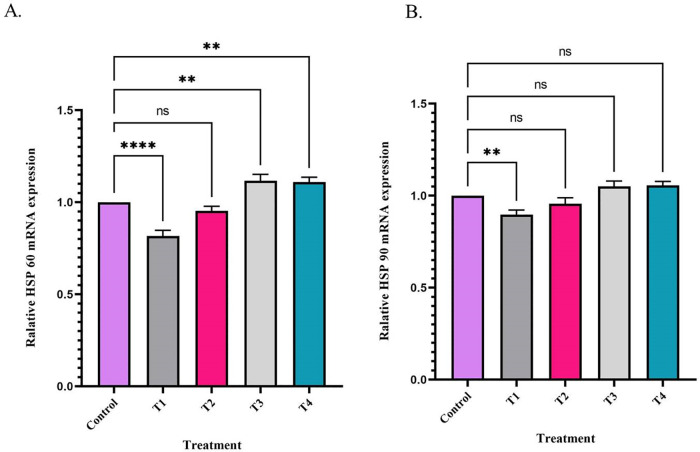
Changes in relative mRNA transcript of antioxidant-related gene in hepatopancreas (A), HSP 70 (B), HSP 90 of *M. rosenbergii* after exposure to different salinities.

mRNA transcripts of the stress response gene (HSP 60 and HSP 90) indicate a significant down-regulated expression in *M. rosenbergii* exposed to 3 ppt, as compared to the control group (**Fig 6A**, **6B**). An increase in rearing salinity to 9 ppt and 12 ppt showed significantly higher HSP 60 mRNA transcript levels relative to the control. Although the expression level of HSP90 moderately increased at 9 ppt and 12 ppt rearing conditions, the differences were not statistically significant from the control group (**Fig 6B**).

### 3.8. Changes in expression of immune-related gene

**Fig 7:**
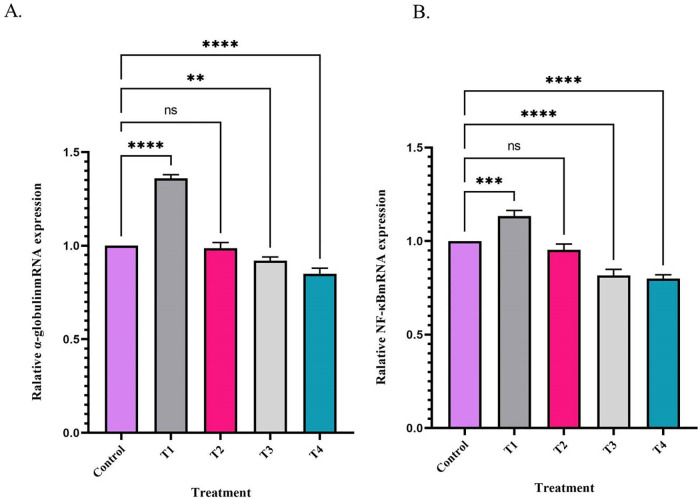
Changes in relative mRNA transcript of antioxidant-related gene in hepatopancreas (A), HSP 70 (B), HSP 90 of *M. rosenbergii* after exposure to different salinities.

The relative expression of immune-related genes (α2 macroglobulin and NF-κB) was significantly up-regulated in *M. rosenbergii* reared at 3 ppt salinity, as compared to the other groups (**Fig 7A**, **7B**). The expression levels of α-2 macroglobulin and NF-κB genes were highest in prawns exposed to 3 ppt salinity, followed by the control group, 6 ppt, and 9 ppt, with the lowest expression observed in prawns from 12 ppt.

### 3.9. Changes in expression of antioxidant-related gene

**Fig 8:**
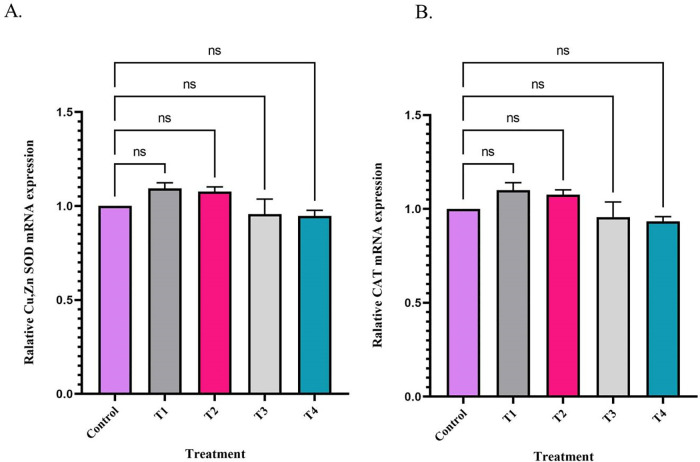
Changes in relative mRNA transcript of antioxidant related gene in hepatopancreas (A), Cu, Zn-SOD (B), CAT of M. rosenbergii after exposure to different salinities.

Expression of the antioxidant related genes (Cu, Zn-SOD and CAT) showed that the genes involved in oxidative stress were up-regulated at 3 ppt salinity, while no significant differences were recorded for the other treatment groups (**Fig 8A**, **8B**).

## 4. Discussion

Salinity played a significant role in the rearing and survival rate of the giant freshwater prawn (*M. rosenbergii*). Among the water quality parameters, optimum salinity was found to be crucial for larval growth, nutrient utilization, and survival of the prawn [40]. Temperature also significantly influenced the growth and survival. Regarding other water quality parameters in the trials, both pH, dissolved oxygen, alkalinity, NH_3_, and NO_2_ remained within the optimal range for freshwater prawn culture throughout the experimental trial [33].

In this study, the maximum survival rate and growth performance were documented at a salinity level of 3 ppt, indicating favorable physiological conditions for this species at this salinity. In contrast to this, the lowest survival and growth rates were observed at 12 ppt, suggesting that elevated salinity levels may impose physiological stress, potentially affecting osmoregulation, metabolism and overall health. A previous study suggested that *M. rosenbergii* can be cultured in salinities up to 10 ppt, but a better production, larger individual size, and higher survival rates were observed at a salinity of 5 ppt [41]. In another study of salinity preferences, [32] observed that *M. rosenbergii* achieved its highest growth rate at 0 ppt salinity, but as the salinity increased, growth rates progressively declined and growth was inhibited at 18 ppt. A reduced growth at high salinity in prawns appears to be due to a decreased appetite and a reduced food assimilation efficiency [42,43].

At high salinities, studies have shown that hyper-osmoregulation in crustaceans requires energy, which is primarily derived from proteins and or lipids [43–45]. In addition to the physiological stress, the growth of *M. rosenbergii* can be adversely affected by elevated salinities due to increased energy expenditure, protein sparing, and depletion of lipid reserves. As a consequence, the biomass of *M. rosenbergii* is reduced relative to rearing in lower salinities [46]. Based on the results of the quadratic regression analysis, the optimal feeding rate should be determined by considering growth parameters, WG (g) and SGR (%/day). Our findings demonstrate that the optimal salinity levels for maximum growth performance and an efficient nutrient utilization are 3.12 ppt for WG and 3.80 ppt for SGR.

Condition factor (CF) is an important tool for evaluating shellfish health, providing insight into the overall well-being of a population by assessing its relative plumpness [47]. Here, we observed the highest CF value for rearing under the 3 ppt, but all CF values obtained in this study indicate that the experimental species were in good condition (**Fig 3**). The observed findings between WG and SGR, relative to FCR is in agreement with the findings of [48]Shi et al. (2024), who reported that FCR is significantly correlated with growth traits.

The application of molecular tools in recent aquaculture research has provided deeper insights into animal responses to culture-related stress and environmental fluctuations [21,22,49]. In this study, an attempt has been made to determine the mRNA expression of growth factors, stress responses, antioxidant enzymes, and immune-related genes under different salinity treatments.

Myostatin, belonging to the transforming growth factor (TGF)-β super family, suppresses the myoblasts differentiation and proliferation and is inversely associated with the growth traits of fish and shellfish [50]. MSTN negatively controls muscle growth by retarding protein synthesis and simultaneously promoting protein degradation, resulting in a decrease in skeletal muscle mass [51]. In our experiments, the expression of the MSTN gene in *M. rosenbergii* was down-regulated when reared under a salinity condition of 3 ppt. Thus, the low level of MSTN expression recorded in *M. rosenbergii* suggests a favorable growth, as it was inversely correlated with growth performance. Similar findings were earlier found for *Litopenaeus. vannamei* [52] and *Fenneropenaeus chinensis* [53], and also for *M. rosenbergii* [54]. Previous studies reported that crustacean hyperglycemic hormone CHH plays a role in multiple biological processes, including growth, molting, reproduction, metabolism, morphogenesis and osmoregulation [55,56]. In this study, CHH expression enhanced the growth performance of *M. rosenbergii* reared at 3 ppt salinity, suggesting that this salinity level stimulates metabolic activity. [57] reported that the expression pattern of CHH was markedly modulated in *L. vannamei* exposed to different salinity conditions

Molting is an important process in crustaceans during their entire growth and development. The molting is significantly influenced by molting inhibitory hormone (MIH) [58]. This enzyme plays a pivotal role by inhibiting the molting of crustaceans by lessening the formation of ecdysteroids [59] and other molting related compounds [60]. In our study, MIH gene expression in *M. rosenbergii* was significantly up-regulated at 12 ppt salinity, compared to the other salinities, indicating physiological stress or altered endocrine regulation, potentially leading to decrease molting activity. It has been reported that MIH inhibits the activity of Y-organs throughout the molting period [61] and that a lowered level of MIH secretion influences the molting in crustacean [62]. [63] have also reported that the mRNA transcript of the MIH gene in *L. vannamei* is significantly influenced by salinity fluctuations. A significantly lower expression of the CHIT gene at 12 ppt salinity indicating that excessive salinity may inhibit expression, potentially negatively affecting the molting and metamorphosis of *M. rosenbergii.* According to previous findings, different isoforms of CHIT showed significant up-regulation in the mud crab (*Macrophthalmus japonicas*) when exposed to different salinities [64]. Thus, chitinase production reflects molting in *M. rosenbergii* and other crustaceans.

HSPs are the prime stress proteins synthesized by organisms and serves as a biological response marker for stress indication [65]. HSPs play an influential role in both innate and adaptive immune activation and responses [66]. In this study, the expression levels of the HSP60 and HSP90 mRNA transcripts were markedly decreased at a salinity of 3 ppt, suggesting a role in maintaining cellular homeostasis. These findings were in accordance with [67], who reported that the expression of different isoforms of HSPs showed down-regulation in the *M. japonicus* after exposure of low salinity stress. In *Portunus trituberculatus*, exposure to low salinity conditions led to the down-regulation of heat shock protein (HSP) gene expression [68].

Antioxidant enzymes play an influential role in mitigating oxidative stress by neutralizing excessive reactive oxygen species (ROS) [69]. It is well-documented that the expression levels of both SOD (superoxide dismutase) and CAT (catalase) are key biomarkers for evaluating the antioxidative defense mechanisms against pathogenic infections in *L. vannamei* [70]. In this study, salinity exposure at 3 ppt in *M. rosenbergii* led to the up-regulation of antioxidant enzyme genes, indicating defensive response by scavenging free radicals.

Crustaceans possess a highly responsive innate immune system that relies on multiple immune -related proteins associated with the prophenoloxidase (proPO) system. Among these proteins, α2-macroglobulin plays a critical role in pathogen defense and immune regulation by inhibiting proteases that trigger the proPO activation [71,72]. The present results indicate a significant increase in α2-M mRNA expression at 3 ppt, suggesting its role in modulating the immune response. NF-κB (nuclear factor kappa B) is a highly conserved transcription factor that plays a crucial role in regulating the expression of downstream genes involved in various cellular processes. In *M. rosenbergii* NF-κB exhibited significantly higher expression levels in the 3ppt salinity group compared to the other groups, suggesting its involvement in stress adaptation and cellular response mechanisms. This was in agreement with the results in previous findings reported by [73]. The upregulation of SOD, CAT, α2-M, and NF-κB at 3 PPT does not indicate a detrimental stress. Rather, it reflects a mild adaptive response, in which low salinity acts as a physiological stimulus. This homeostatic effect enhances antioxidant and immune functions while maintaining optimal growth and survival, indicating a balanced distribution of energy between growth and defense. Furthermore, at higher salinities (9 and 12 ppt), the upregulation of stress-responsive genes and downregulation of immune-related genes may reflect increased osmoregulatory and physiological stress. These changes could be associated with reduced energy allocation to immune function, thereby contributing to growth inhibition and decreased survival rates.

## 5. Conclusions

Salinity gradient has been inconsistent gradually affecting growth, reproduction, and other physiological traits in fish and invertebrates. The findings of the present investigation recommended that 3 ppt salinity is optimum for substantial growth, feed utilization, and other physico-biochemical profiles in *M. rosenbergii*. However, exploration of the effects of salinity gradient on enzymatic and non-enzymatic antioxidant assay, and transcriptomic analysis is a warrant for future studies.

## Supporting information

S1 Table Concentration, and purity of RNA isolated from the hepatopancreas of M. rosenbergii (n = 3) under different salinity treatment. (DOCX)
