# Supplementary material for: Influence of salinity on growth, nutrient utilization, and gene expression of giant freshwater prawn (Macrobrachium rosenbergii)
Source: PLoS One. 2026 Jul 23;21(7):e0353240. doi: 10.1371/journal.pone.0353240 (PMC13395321; doi:10.1371/journal.pone.0353240)
Supplement: S1 Table — (DOCX) [file pone.0353240.s001.docx]

| Treatment | RNA concentration (ng/µl) | A260/280 | A260/A230 |
| --- | --- | --- | --- |
| Control | 750.2±10.20 | 2.05± 0.20 | 2.10± 0.14 |
| T1 | 910.8± 10.80 | 2.01± 0.15 | 2.08± 0.10 |
| T2 | 690.3±10.26 | 2.06± 0.18 | 2.12± 0.17 |
| T3 | 820.5±8.27 | 1.98± 0.10 | 2.00± 0.2 |
| T4 | 765.4±10.26 | 2.02± 0.10 | 2.05± 0.16 |

**Supplementary Table 1**
